# Recent advancement in the anticancer efficacy of the natural flavonoid scutellarin: a comprehensive review

**DOI:** 10.3389/fphar.2025.1579609

**Published:** 2025-03-27

**Authors:** Deena Elsori, Pratibha Pandey, Meenakshi Verma, Nasir Vadia, R. Roopashree, Manish Vyas, L. Lakshmi, Laxmidhar Maharana, Deepak Nathiya, Mohd Saeed, Safia Obaidur Rab, Fahad Khan

**Affiliations:** ^1^ Faculty of Resilience, Rabdan Academy, Abu Dhabi, United Arab Emirates; ^2^ Centre for Research Impact and Outcome, Chitkara University Institute of Engineering and Technology, Chitkara University, Rajpura, Punjab, India; ^3^ University Centre of Research and Development, University Institute of Biotechnology, Chandigarh University Gharuan, Mohali, Punjab, India; ^4^ Department of Pharmaceutical Sciences, Marwadi University Research Center, Faculty of Health Sciences, Marwadi University, Rajkot, Gujarat, India; ^5^ Department of Chemistry and Biochemistry, School of Sciences, JAIN (Deemed to be University), Bangalore, Karnataka, India; ^6^ School of Pharmaceutical Sciences, Lovely Professional University, Phagwara, Punjab, India; ^7^ Department of Nursing, Sathyabama Institute of Science and Technology, Chennai, Tamil Nadu, India; ^8^ Department of Pharmaceutical Sciences, Siksha 'O' Anusandhan (Deemed to be University), Bhubaneswar, Odisha, India; ^9^ Department of Pharmacy Practice, NIMS Institute of Pharmacy, NIMS University Rajasthan, Jaipur, India; ^10^ Department of Biology, College of science, University of Hail, Hail, Saudi Arabia; ^11^ Department of Clinical Laboratory Sciences, College of Applied Medical Sciences, King Khalid University, Abha, Saudi Arabia; ^12^ Center for Global Health Research, Saveetha Medical College and Hospital, Saveetha Institute of medical and Technical Sciences, Chennai, Tamil Nadu, India

**Keywords:** scutellarin, natural flavonoids, cancer, formulations, signaling pathways

## Abstract

Scutellarin (SC), a natural flavonoid, has been expansively employed in treating innumerable inflammation-related diseases due to its antitumor, antiinflammatory, anticancer, and antioxidant potential. Scutellarin can inhibit significant inflammatory cell signaling pathways, comprisingPI3K/Akt, NF-κB, and MAPK, and while activating antioxidant-related pathways such as Nrf2 and ARE. Numerous reviews have outlined scutellarin’s pharmacological effects and associated mechanisms in inflammation-related diseases. Several studies have elucidated the mechanisms of anticancer activity by inhibiting various signaling pathways; however, to our knowledge, none of the reviews have distinguished the anticancer potential of scutellarin based on different human cancer types. Our review outlined detailed insights about the anticancer potential of scutellarin based on cancer type in the human body. Furthermore, we have also outlined formulations, combinatorial therapies, and comprehensive mechanistic research to deliver enhanced and effective treatment options for cancer patients. This study will provide thorough and detailed insights into scutellarin, supporting its development as a promising candidate for cancer treatment.

## 1 Introduction

Flavonoids are polyphenolic bioactive secondary metabolites synthesized by plants, contributing to their flavor, color, and pharmacological attributes (Roy et al., 2022). Flavonoids safeguard plants from detrimental environmental influences and have garnered interest for usage in several experimental and epidemiological investigations aimed at assessing their potential therapeutic advantages for various acute and chronic human ailments (Bolat et al., 2024; Jain et al., 2019). Numerous experimental investigations have proven their significant anticancer (Kopustinskiene et al., 2020), immunomodulatory (González-Gallego et al., 2014), and anti-inflammatory properties (Han L. et al., 2022; EghbaliFeriz et al., 2018). Increasing evidence indicates that many flavonoids possess anticancer activities, while the molecular mechanisms remain inadequately elucidated. The genus *Scutellaria* is rich source of plant derived components, including flavonoids, terpenoids, steroids, alkaloids, and phenols (Nourizadeh, 2022).

**FIGURE 1:**
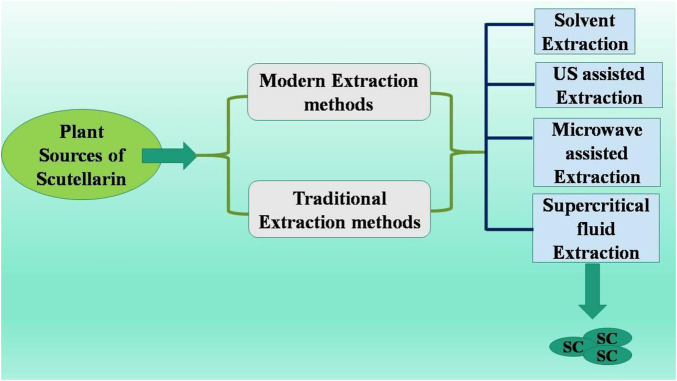
Methods to obtain scutellarin (Vesaghhamedani et al., 2023; Yang et al., 2019a; Xiang and Wu, 2017; Yang and Wei, 2018; Yang et al., 2019b; Hao et al., 2020).

Scutellaria is an ethnobotanical herb from the Lamiaceae family, utilized to treat various diseases, including cancer, cirrhosis, neurological problems, jaundice, anxiety, and hepatitis (Irvin et al., 2019). In Chinese, *Scutellariae Barbatae Herba*, or Ban Zhi Lian, serves as a chemical marker for quality control purposes (Shen et al., 2021). Scutellarin (flavonoid glycoside) is predominantly found in the genera *Erigerontis* and *Scutellaria* (Jiang et al., 2014). Scutellarin is commonly found in *Scutellaria baicalensis* and has also been identified in *Oroxylum indicum* (L.) Kurz., *Scutellaria lateriflora* L., and *Scutellariabarbata*D.Don (Jia et al., 2024). The Scutellaria genus is extensively found in North America and East Asia. The foliage, roots, and stems of these plants possess substantial quantities of scutellarin. Breviscapine, an extract from *E. brevisacapus* (Vaniot) *Hand.-Mazz.,* abundant in scutellarin, has been documented to enhance blood circulation (Zhou et al., 2024a). Research indicated that SC concentration varied considerably based on plant species and its respective portions. Scutellarin is the main active substance (not only in Erigeron plants) but also widely distributed in *Centaurus, Scutellaria, Patrinia villosa, Opuntia, Conya sumatrensis, Perilla frutescens, Anaphalis, Conyza canadensis, Scutellariabaicalensis, Erigeron breviscapus, Centaurea montana, Anaphalis sinica, Rosmarinus officinalis, Scutellariabarbata, Erigeron multiradiatus, Thymus mongolicus, Vernonia esculenta,* and *Juniperus rigida* (Zhou et al., 2024a; Vesaghhamedani et al., 2023). SC extraction methods include traditional extraction as well as modern extraction techniques (Figure 1). Vesaghhamedani et al., have explained in detail about the sources of SC and its extraction methods in their review report (Vesaghhamedani et al., 2023). For instance, roots (from *S. baicalensis*) and herb of *Erigeron brevisacapus* are major sources used in SC extraction. There are several extraction methods being used for SC extraction such as solvent extraction, Ultrasonic (US) assisted extracted, microwave assited extraction, and supercritical fluid extraction. Solvent extraction method utilize infusion process for extraction with low equipment requirement but with the limitation of lower extracting efficiency and more time consuming process (Yang et al., 2019a). US assisted method utilizes cavitation effects of US waves with high extraction efficiency and less time consumption. But this method needs more specialized US extraction equipment (Xiang and Wu, 2017). Microwave assistant extraction method involves usage of microwave energy to promote the release of SC with higher extraction speed and less energy consumption. Again this method also requires more specialized microwave extraction equipment. Supersritical fluid extraction method utilizes supercritical CO_2_ as a solvent for SC extraction with higher efficiency and environment friendly process. However this method also requires expensive and complicated processes and equipments (Yang and Wei, 2018; Yang et al., 2019b).

Numerous researches have evidenced the anti-cancer properties of scutellarin in colon, esophagus, bladder, and breast cancers (Xie et al., 2024). Scutellarin’s anticancer action is attributed to its capacity to inhibit multiple cell signaling pathways, including JAK/STAT3, NRF2, AMPK/ERK, MAPK, and β-catenin/Wnt (Xie et al., 2024). Moreover, SC stimulates both the types of apoptotic (intrinsic and extrinsic) pathways, resulting in tumor cell death, disruption of the cell cycle, and facilitation of cell cycle arrest. Scutellarin diminishes tumor aggressiveness by inhibiting treatment resistance, metastasis, angiogenesis, and other carcinogenic processes (Zhou et al., 2024b). Scutellaria possesses extensive pharmacological potential, encompassing arachidonate metabolism, anti-allergic properties, diuretic effects, antioxidant activity, laxative functions, lipid regulation, analgesic capabilities, anti-diabetic effects, and anti-inflammatory properties (Lai and Li, 2024a). The clinical development of scutellarin is hindered by a number of issues, including poor pharmacokinetic characteristics, bioavailability, and solubility, despite its encouraging anticancer efficacy (Wang and Ma, 2018). Consequently, it has been proposed that specific alterations can improve scutellarin’s pharmacogenetic properties and lessen its restricted water solubility. This review study aims to investigate its clinical utility and enhance its therapeutic potential in subsequent parts. This review will examine all facets of the medical benefits of scutellarin, as well as its limits.

## 2 Pharmacokinetic studies of SC and bioavailability of scutellarin

Experimental (*In vivo*), pharmacokinetic investigations showed that SC has certain limitations in its therapeutic effects despite its high anti-inflammatory effectiveness in numerous cellular trials. According to Guo et al. (2021), scutellarin’s oral bioavailability in Beagle dogs reported 0.40% ± 0.19%, meaning it was barely absorbed (Guo et al., 2021). Additionally, scutellarin has a short elimination half-life of 52 ± 29 min and is quickly metabolized and eliminated following intravenous injection (Feng et al., 2017). Furthermore, because scutellarin’s chemical structure contains a phenolic hydroxyl group, precipitation in an acidic solution will be quickly produced (Song et al., 2020). According to research, flocculent precipitation may happen when scutellarin dissolves in an infusion solution with a pH below 3.8 (Xing et al., 2011a). This suggests that the gastrointestinal system absorbs less of it, which helps to explain why its bioavailability is limited. A growing number of contemporary technologies have been progressively implemented to enhance scutellarin’s efficacy and solve the issue. Various specialized materials are utilized to enhance the dose form. Triglyceride mimetic prodrug of SC showed improved oral bioavailability and intestinal lymphatic transport (Wang X. et al., 2021); Liposome technology was employed to prepare liposome precursors of SC that its displayed enhanced stability and bioavailability (Ma et al., 2023). For instance, β-cyclodextrin suspension polymers employed as carriers improved SC solubility (Wang J. et al., 2021).

Additionally, novel dosage forms, including encapsulating technology, fat emulsion, and self-microemulsion, have been extensively utilized in exploring SC formulations, bioavailability enhancement, better solubility and safety (Tian et al., 2016). Pharmacological targeting of scutellarin was examined using multiple approaches. In cerebrovascular disorders, the blood-brain barrier significantly obstructs therapeutic efficacy, prompting the emergence of nasal delivery as a viable alternative (Zeng et al., 2024). *In situ* gels derived from nanosuspensions were formulated for intranasal delivery of SC, demonstrating enhanced solubility, bioavailability, and extended retentivity in nasal cavity (Chen et al., 2020). SC nanoformulations utilizing PLGA-PEG-AEAA also been engineered to enhance tumor delivery (Li et al., 2023). Additionally, a conventional coating technique has been implemented for enhancing drug targeting. SC also got formulated as a coated tablet designed for colon localization, with drug release occurring at a pH greater than 7.0, enhancing colon-targeted drug delivery (Li Y. L. et al., 2013). Cremophor EL (at its nontoxic doses) enhanced SC transportation by MRP3 and inhibited SC efflux transportation by MRP2 and BCRP concurrently (Li and Liu, 2024). It also exhibited a strong capacity to inhibit MRP2 and BCRP while efficiently activating MRP3. Additionally, pharmacokinetic studies in rats also validated enhanced oral SC absorption by Cremophor EL (Xiao et al., 2016). Another study reported that compared with the normal group, the MRT of scutellarin was significantly increased, which demonstrated that the pathological state of hepatic injury might prolong its residence time in the human body. PK/PD modeling analysis suggested that the plasma drug concentration of scutellarin had a good correlation with the three AST, ALT, and LDH, and the lag time of the efficacy of scutellarin is relatively long (Zhu et al., 2023). A recent review has elaborated the pharmacological efficacies and pharmacokinetics of scutellarein (Lai and Li, 2024b). Till date, only one clinical trial with this compound has been successful with breast cancer patient (ClinicalTrials.gov ID - NCT00028977). Both the phases (I and II) are completed. This was a pilot study that was conducted to assess the efficacy and feasibility of *Scutellaria barbata* herb for the treatment of metastatic breast cancer patient.

Scutellarin exhibited limited absorption (Xing et al., 2011b), which impeded it’s *in vivo* utilization and thus modifying scutellarin using biological or chemical methods is advisable to produce derivatives with enhanced bioavailability and improved solubility (Xing et al., 2011c). The hydrophobic cavity of cyclodextrins, truncated-cone polysaccharides, can accommodate a variety of chemical and biological substrates (Poulson et al., 2021). Given that the cavity contains accessible surface molecules, these characteristics might enable it to stick to the surface of tissues or cells. Therefore, the conjugates are used for drug delivery, covalently attaching drug molecules to cyclodextrin (Crini, 2014; Laza-Knoerr et al., 2010). Three SC cyclodextrin conjugates such as enamino-SC-β-cyclodextrin, dienamino-SC-β-cyclodextrin, and amino-SC-β-cyclodextrin were prepared to assess their anticancerpotential and solubility on colon cancer cells (HT-29, SW480, HTC116, and LoVo) (Yang et al., 2013; Liao et al., 2020). In addition to mono-cyclodextrin SC conjugation, cyclodextrin-based polyrotaxane has also been proposed for conjugation of SC. Polyrotaxane (mechanically interlocked molecule) consisting of linear chains and cyclic structures, wherein numerous rings are threaded over molecular axle. Polyrotaxanes derived from cyclodextrin are increasingly favored for their wider potential in pharmaceutical sector (Petitjean et al., 2021). SC-polyrotaxane combination was synthesized and exhibited superior antiproliferative efficacy on human cancer (HCT116 and LoVo) cells compared to free SC and SC cyclodextrin, indicating its impending utility in treating human colon carcinoma (Jiang et al., 2013).

The scutellarin-cyclodextrin combination was additionally modified with folic acid (FA) to augment tumor-targeted treatment (Qiu et al., 2017; Liao et al., 2015). FA has been widely utilized as potent targeting ligand in recent years since folate receptor is overexpressed in numerous human carcinomas including kidney, colon, breast, ovary, rectum and lung while exhibiting meek expression in normal tissues (Xia and Low, 2010; Ebrahimnejad et al., 2022). As a result, several pharmacological carriers incorporating folic acid have been examined (Narmani et al., 2019). Cyclodextrin has demonstrated the capability of conjugating with FA to target folate receptors on neoplastic cells (Xu et al., 2016). Bioconjugation of FA to β-cyclodextrin through cationic polyamine spacer has produced an efficient therapeutic carrier for scutellarin, a weakly soluble therapeutic molecule, by utilizing the hydrophobic cavity of cyclodextrin to target cancer cells (Yuan and Li, 2017). Supramolecular drug carrier formed by FA bioconjugation, β-cyclodextrin, and polyamine enhanced thermal stability, bioavailability, solubility of SC, resulting in increased cytotoxicity of this complex relative to SC alone in both cancer (HCT116 and LoVo) cells (Liao et al., 2015).

Nitric oxide (NO) has garnered significant attention as prospective anticancer agent in past decade. A multitude of molecular mechanisms facilitate NO-induced cell death (Huang et al., 2017; Huang et al., 2022). Generally, increased nitric oxide concentrations can induce apoptosis, inhibit metastasis, and enhance the vulnerability of tumor cells to immunotherapy, chemotherapy, and radiation (Li et al., 2022). Nitrate and furoxan can generate substantial NO production (both *in vitro* and *in vivo*) that are frequently utilized in pharmacology (Mondal et al., 2024). Consequently, NO-releasing hybrids development represented viable and promising therapeutic strategy for carcinoma (Parisi et al., 2024). Using these notions, novel SC derivatives were synthesized employing diverse linkers and furoxan or nitrate moieties, followed by an evaluation of their anti-proliferative properties on human tumor cell lines PC3, MCF-7, HCT-116, and HepG2, as well as L-O2 (normal liver cells) (Gunter and Mah, 2023). The modifications in scutellarin enhanced its anti-proliferative effects on tumor cells while exhibiting low toxicity towards L-O2 cells, indicating that the NO-scutellarin hybrid possesses favorable selectivity between normal and malignant cells (Han et al., 2017).

Scutellarin has undergone additional changes to improve its bioavailability. Ni et al. synthesized several long aliphatic-chain scutellarin derivatives and investigated their anti-proliferative properties on the cancer cells Jurkat, MDA-MB-231, and HCT-116 (Iqbal et al., 2017; Ni et al., 2018). Suitable long aliphatic chain enhanced anti-proliferative action of SC, as one of the synthesized derivatives exhibited more significant anti-proliferative potential on HCT-116 and Jurkat cells (Gharari et al., 2022). Amide groups were employed alongside long aliphatic chains to modulate the activity of scutellarin (Cao et al., 2005). Derivatives of amide-scutellarin demonstrated potent anti-tumor properties against two human leukemia (HL-60 and THP-1) cells, similar to long aliphatic chains. These amide-conjugated SC complexes demonstrated neuroprotective properties in addition to their anti-tumor potential, which could reduce neurotoxicity of chemotherapeutic drugs in cancer treatment regimens (Han et al., 2022b; Han et al., 2022c). Scutellarin was used as an anticancer medication, polymer cyclodextrin (PCD) as a molecular switch, and core-shell structure of Fe_3_O_4_ mesoporous silicon (MSN-Fe_3_O_4_) as the main body to create a pH/H_2_O_2_ dual-responsive and targeted nanocarrier system (NCS) (El-Sayed et al., 2024). NCS exhibited more significant cytotoxicity against tumor (Huh7 and HCT116) cells owing to its dual-triggered response to pH and H_2_O_2_, whereas PCD-MSN-Fe_3_O_4_ showed reduced cytotoxicities against both cancer (HCT116 and Huh7) cells (Hossen et al., 2019). In mice subcutaneous tumor models, *in vivo* therapeutic study of NCS shows a considerable suppression of tumor growth with no discernible adverse effects. The NCS improved SC bioavailability and employed magnetic targeting technology to administer SC to tumor locations precisely. These outcomes highlighted the significant therapeutic applicability of NCS (Yi et al., 2024).

## 3 Anticancer potential of scutellarin and their mechanism

Scutellarin exhibited multifaceted anticancer efficacies, including apoptotic induction, inhibition of cancer cell growth, metastasis suppression, and angiogenesis inhibition, altered tumor cell’s metabolism, modulated inflammation and altered immune responses and noncoding RNAs expression (EghbaliFeriz et al., 2018; Shi et al., 2015). These efficacies are mediated via various cell signaling pathways and molecular targets in numerous human carcinomas. While numerous researches have elucidated the mechanisms underlying the anticancer potential of scutellarin, we have sought to differentiate its anticancer efficacy based on its various forms within the human body rather than its mechanisms (Li-Weber, 2009). This review would definitely provide a specific insight to those researchers who are dealing with natural carcinomas and specific human carcinomas for developing better chemotherapeutic approach. Further subsections would start with different human carcinomas against which scutellarin has been found to be safe and effective. Baicalin, wogonin and baicalein are the main components of *S. baicalensis*. These phytochemicals exhibit both cytostatic and cytotoxic properties against diverse human cancer cells (*in vitro*) and impede tumor growth (*in vivo*) (Li-Weber, 2009). Crucially, they exhibit negligible or minimal toxicity to epithelial (normal) cells, as well as to peripheral blood (normal) and myeloid cells. In rodents, the acute toxicity data revealed that the no-observed bad effect level was 2,250 mg/kg, while the lowest-observed adverse effect level was 3,375 mg/kg; also, there was no treatment-related death among the animals in the acute toxicity research. No mortality was seen in rats administered scutellarin at dosages of 3, 5, 7, and 10 g/kg; hence, scutellarin was well tolerated at doses above 10 g/kg. The LD_50_ value of scutellarin was undetectable from the oral test doses; consequently, a maximum tolerated dose experiment was conducted, revealing that the maximum tolerated dose exceeded 10 g/kg, indicating that scutellarin can be classified as non-toxic upon acute ingestion (Li et al., 2011).

Anticancer properties of these flavones mostly stem from their capacity to scavenge oxidative radicals, diminish NF-κB activity, inhibit various genes critical for cell cycle regulation, lower COX-2 gene expression, and avert viral infections (Almatroodi et al., 2021). In clinical trials, Scutellaria has been evaluated as a therapeutic or adjunctive therapy for several breast and prostate malignancies, with little toxicity across different dosage forms (Sun et al., 2024).

### 3.1 Renal cell carcinoma

Apoptosis is a systematic and regulated process that facilitates cell death (Kim-Campbell et al., 2019). Chemotherapeutic agents induce apoptosis in tumor cells, and deficiencies in apoptotic pathways are critical in carcinogenesis and developing resistance to cancer treatment (Kaufmann and Earnshaw, 2000; Makin and Dive, 2001). Apoptotic abnormalities might lead to enhanced proliferation of tumor cells. Cell signaling exhibits a dual role in tumor cell formation. The induction of apoptosis through the regulation of signaling pathways is a mechanism by which anticancer therapies exert their effects (Yaacoub et al., 2016). The cell cycle consists of events that enable cell proliferation and growth, with cyclins, cyclin-dependent kinases (CDKs), and CDKIs serving as essential components. Cells with overexpressed cyclin levels or reduced CDK inhibitors demonstrated uncontrolled proliferation and cell cycle dysregulation associated with cancer. Consequently, mechanisms or agents inducing cell cycle arrest in cancer cells have garnered significant attention in recent research (Ding et al., 2020). SC significantly induced cell cycle arrest (G0/G1 phase) and apoptosis and in RCC (at 30, 60, and 90 μM dosage) cells. This effect is accompanied by a notable reduction in key proteins, including Bcl2, cyclin D1, MMP-9, CDK2, and MMP-2 alongside an increase in p21, Bax, and cleaved caspase 3 (Guo et al., 2023).

**FIGURE 2:**
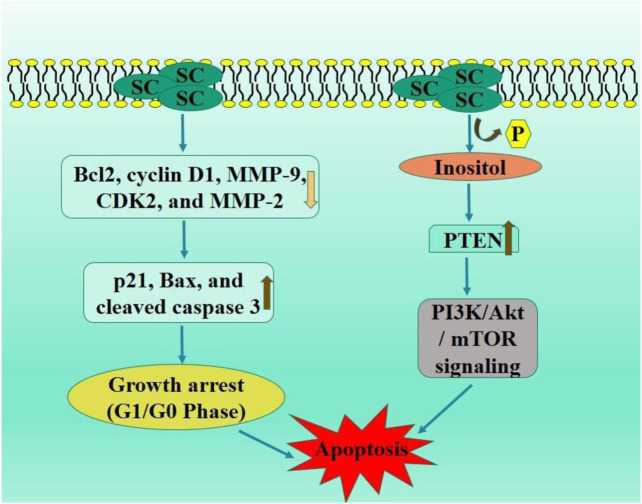
Diagrammatic illustration by which scutellarin induce apoptosis and growth arrest in renal cell carcinoma.

PI3K/Akt signaling pathway is mediator and enhancer of epithelial-mesenchymal transition and metastasis in tumorous cells (He H. et al., 2021). PTEN is a tumor suppressor protein, frequently mutated in various cancers, leading to the inhibited PI3K/Akt pathway via catalyzing inositol dephosphorylation on plasma membrane. PTEN exhibits anti-tumor efficacies in malignancies, including gliomas, colorectal, and non-small cell lung carcinoma (Luongo et al., 2019; Milella et al., 2015). The *in vivo* assay demonstrated that SC exhibited no toxic effects, significantly elevated the PTEN levels, and inhibited activation of PI3K/AKT/mTOR signaling pathway. Ectopic PTEN expression increased inhibitory effect of SC on RCC proliferation, whereas PTEN knockdown negated this effect by modulating the downstream P13K/AKT/mTOR signaling pathway (Deng et al., 2018) (Figure 2).

### 3.2 Colorectal cancer

Colorectal cancer (CRC) arises from mutations affecting genes associated with DNA repair pathways, oncogenes, and tumor suppressor genes. Recent genomic methodologies have facilitated the identification of numerous genetic abnormalities associated with colorectal cancer (Deng et al., 2018). Consequently, whereas mutations represent the primary genomic alterations, other chromosomal abnormalities and translocations are also commonly observed in colorectal cancer (CRC) (Alzahrani et al., 2021). These abnormalities impact critical pathways (WNT, MAPK/PI3K, TGF-β) and cellular processes (TP53 and cell-cycle control). Modifications impacting these pathways thus provide proliferative benefits to tumor cells. Tumor-induced angiogenesis has become a compelling target for anti-cancer pharmacotherapy (Armaghany et al., 2012). SC has been discovered to influence AKT signaling pathways by modulating the angiogenesis of malignant cells (Al-Ostoot et al., 2021). In the angiogenesis process, new blood vessels in the tumor microenvironment were activated and moved into new capillaries through growth factors and signaling pathways. Inhibiting tumor cell-induced angiogenesis enhances the probability of postponing the malignant advancement of cancer. SC inhibited viability of colorectal cancer cell and colony formation and significantly diminished tumor growth in animal xenografts (Li et al., 2021). SC decreased angiogenesis and metastasis in colorectal cancer by blocking ephrinb2 signaling, thereby providing a compelling justification for its utility in suppressing colorectal cancer metastasis and angiogenesis. In HCT-116 cells, SC administration diminished viability and elicited apoptotic alterations, as well as modulated Bcl-2/Bax gene expression levels. This results in an elevation in caspase-3 protein production and p53 phosphorylation in HCT-116 cells. Moreover, p53 inhibition with pifithrin-α (a particular inhibitor) negated the pro-apoptotic effects of SC in HCT-116 cells. SC decreased the viability and triggered apoptosis in human colon cancer cells, likely viap53 and Bcl-2/Bax gene modulation level (Zhu et al., 2017).

Ephrins facilitate angiogenesis in both healthy and pathological contexts, including cancer-related angiogenesis (Yang N. et al., 2017; Talia et al., 2023). Ephrinb2 and ephb4 significantly modulates VEGF signaling pathway, influencing position of endothelial cells in angiogenesis (arterial and venous) (Papadakos et al., 2023). Ephrinb2 modulates the synthesis of VEGFR, including VEGFR3, through both forward and reverse signaling pathways (Brkić, 2017). In HCT-116 and RKO cells, scutellarin prevented migration, enhanced apoptosis, and significantly decreased the volume and development of colorectal tumors in nude mice without adverse effects on liver function or blood circulation (Du et al., 2020). Additional *in vitro* experiments demonstrated that scutellarin inhibited cell proliferation, transformation, and differentiation of HT-29 CSC cells, significantly down-regulating the mRNA levels of Lgr5, c-Myc, CK20, and Nanog, as well as the protein expression levels of Gli1, and Lgr5 in HT-29 CSC cells (Xiong et al., 2020). Furthermore, animal studies demonstrated that SC markedly suppressed the proliferation of subcutaneous xenografts in nude mice while concurrently down-regulating the mRNA expressions of CK20, Ptch1, c-Myc, CD133, Lgr5, Ki-67, and Gli1 as well as the protein levels of CD133, c-Myc, Ki-67, Gli1, Lgr5, and in the xenografts of nude mice (Lei et al., 2020). Collectively, SC may impede the development of colonic cancer stem cells, likely through the downregulation of hedgehog signaling pathway activity.

Dysregulated Wnt/β-catenin signaling system, which is evolutionarily conserved, governs gene expression and cell invasion, migration, proliferation, and differentiation in colorectal cancer development and progression (Zhao et al., 2022; Zhu and Li, 2023). Scutellarin suppressed carcinogenesis of colitis-associated colorectal cancer (CAC) in mice induced by azoxymethane, resulting in reduced pathological signs. Scutellarin reduced mouse serum levels of TNF-α and IL-6, increased Bax expression, and decreased Bcl-2 levels in the CAC tissues of mice via downregulated Wnt/β-catenin signaling pathway. In CRC HT-29 cells, SC inhibited growth and migration, triggered apoptosis, increased Bax expression, and reduced Bcl-2 levels, likely due to suppressing Wnt/β-catenin signaling in HT-29 cells (Zeng et al., 2021).

Accumulating evidence has demonstrated that the activated hedgehog signaling system and chronic intestinal inflammation are crucial in the development of CAC (Geyer and Gerling, 2021). Scutellarin markedly improved azomethane oxide/sodium dextran sulfate-induced colorectal cancer in mice and promoted apoptosis in the cancerous tissues via suppressing NF-κB-mediated inflammation and hedgehog signaling pathway. SC modulated intricate inflammatory networks in mice CAC and inhibited growth, migration, and colony formation of SW480 cells while inducing apoptosis via downregulated hedgehog signaling pathway. Furthermore, SC reduced the NF-κB-mediated inflammatory response in TNF-α-stimulated IEC-6 cells by diminishing the hedgehog signaling cascade (Zeng et al., 2022). Scutellarin effectively improves CAC via inhibiting hedgehog signaling pathway activity, highlighting its potential utility for CRC in clinical contexts.

**FIGURE 3:**
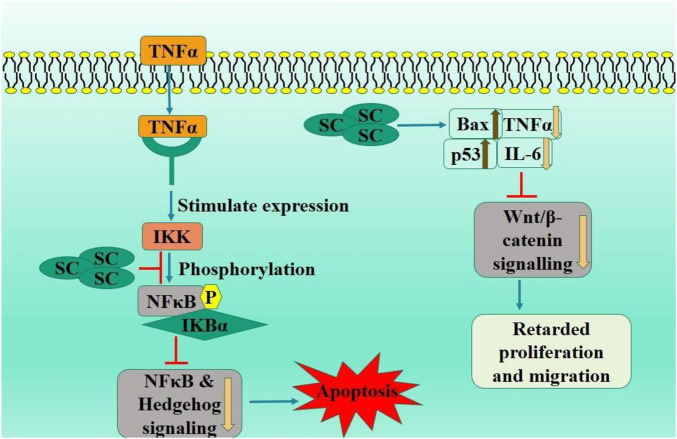
Diagrammatic illustration by which scutellarin induce apoptosis and growth arrest in colorectal carcinoma.

Immune checkpoint inhibitors have transformed cancer treatment (Pandey et al., 2022). However, they have inconsistent efficacy, the potential for relapse, and the capacity to provoke autoimmunity. TNFR2 (signaling protein) located on surface of specific group of Tregs can stimulate proliferation of these cells via NF-κB (Yang et al., 2018). TNFR2 is prominently expressed on surface of numerous malignant malignancies. There is growing evidence that TNFR2 suppression can augment anti-tumor immune responses (Yang et al., 2021). A study examined the inhibitory capacity of TNF-TNFR2 from a chinese herbal extracts and discovered that treatment with this extract might suppress TNFR2-induced biological responses, including TNFR2+Tregs proliferation. Their further work revealed that SC disrupts the connection between TNF and TNFR2, inhibiting p38 MAPK phosphorylation, a downstream signaling component of TNFR2. Significantly, *in vivo* treatment with SC substantially improved the effectiveness of tumor immunotherapy utilizing CpG oligodeoxynucleotide in CT26 colon cancer mice model. Effects of SC correlated with reduction in tumor-infiltrating TNFR2-expressing Tregs and an increase in tumor infiltration of interferon-γ-producing CD8+ T cells. SC or its derivatives could serve as potent adjuvant to augment the anti-tumor efficacy of immunotherapeutic agents via inhibiting TNFR2+ Treg activity (Chen et al., 2022) (Figure 3).

### 3.3 Breast cancer

Advanced breast carcinoma with distant organ metastasis is deemed incurable with existing treatment options (Thill et al., 2018). Breast cancer (at molecular level) is a heterogeneous malignancy characterized by HER2 (encoded by ERBB2), activated hormone receptors (estrogen and progesterone receptor), and/or BRCA mutations (Testa et al., 2020). Therapeutic approaches varies according to molecular subtype and encompasses surgical and radiation therapeutics. Systemic therapies encompassesanti-HER2 therapy for HER2-positive malignancies, endocrine therapy for hormone receptor-positive malignancies, chemotherapy, poly (ADP-ribose) polymerase inhibitors, and immunotherapy (Asgari-Karchekani et al., 2022). SC has been reported to induce apoptosis and impede metastasis, inhibiting cancer proliferation. Consequently, SC may inhibit breast cancer progression, hence enhancing patient survival rates and reducing the incidence of breast cancer-related fatalities. A study assessed the impact of SC water extract (SCW) and SC on BCSCs and examined their potential therapeutic benefits on breast cancers in mice. Both SCW and SC reduced the sphere, viability, migration, proliferation, and colony formation of BCSCs. In mice with tumors originating from naïve BCSCs, scutellarin markedly decreased stem cell markers (CD44) expression, tumor growth, proliferation (Ki67) and lung metastasis. Western blot results indicated the participation of PTEN/Akt/mTOR, NF-κB, and Wnt/β-catenin signaling pathways in the inhibitory actions of scutellarin (Ma et al., 2024). Thus, *S. barbata* water extract might have the potential to be further utilized as an adjuvant therapy for reducing the recurrence of breast carcinoma. In a separate *in vivo* investigation, SC diminished the metastasis of TNBC cells and mitigated tumor-associated vascular endothelial barrier damage via inhibited TNFα-induced vascular endothelial barrier disruption, thereby restoring the diminished expression of junctional proteins via modulated TNFR2-ERK1/2-EZH2 signaling pathway (Mei et al., 2022).

**FIGURE 4:**
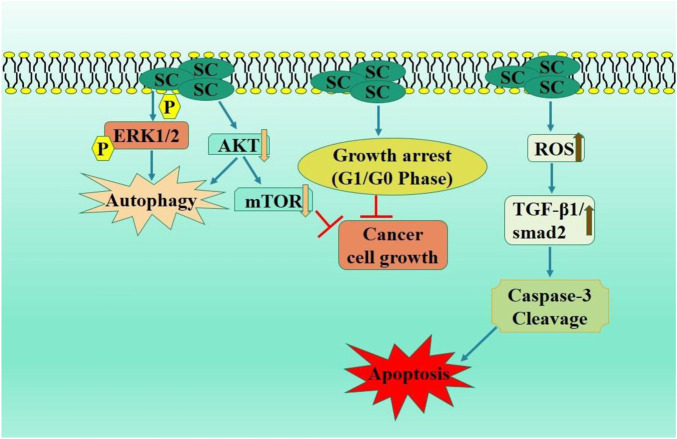
Diagrammatic illustration by which scutellarin induce apoptosis and growth arrest in breast carcinoma.

SC-treated MCF-7 cells exhibited reduced cell proliferation and growth inhibition. Scutellarin markedly suppressed MCF-7 xenograft tumor proliferation, correlating with elevated p-YAP levels and reduced YAP expression. SC treatment of MCF-7 cells potentially promoted apoptosis, which is linked to autophagy induction through the modulation of the HIPPO-YAP signaling pathway, hence supporting therapeutic application of SC-based therapies for improved outcomes in breast cancer patients (Hou et al., 2017) (Figure 4). Therefore, scutellarin may serve as a promising lead candidate for breast cancer therapy through further *in vivo* and clinical studies.

### 3.4 Non-small cell lung cancer (NSCLC)

NSCLC is a diverse category of lung carcinoma mainly caused due to tobacco and smoking; however, radon exposure and air pollution also contribute significantly (Gridelli et al., 2015; Goldstraw et al., 2011). Multiple diagnostic modalities are available for NSCLC, including PET imaging, X-ray, histological analysis, CT of tumor specimens (Herbst et al., 2018). Numerous therapeutic strategies are evolving to target these mutations in EGFR and ALK to address the issue of acquired resistance (Cooper et al., 2022). Palliative care is crucial in patient management and significantly enhances quality of life. SC dramatically decreased cell proliferation in NSCLC cells, activated autophagy, and prompted apoptosis. SC decreased p-AKT expression, while MK-2206, an AKT inhibitor, prompted autophagy. *In vivo* studies using xenograft nude mice shown that SC therapy markedly inhibited p-AKT and tumor proliferation while enhancing p-ERK1/2 and LC3-II expression levels in tumors of mice (Sun C. Y. et al., 2018). In another study, SC significantly inhibited the growth of A549 carcinoma cells, induced substantial G0/G1 phase arrest, enhanced apoptosis and caspase activation. Additionally, SC therapy diminished the levels of p-STAT3, pan-AKT, phosphorylated (p)-mTOR, mTOR, BCL-XL, and STAT3 while elevating the level of 4EBP1 (Cao et al., 2019).

**FIGURE 5:**
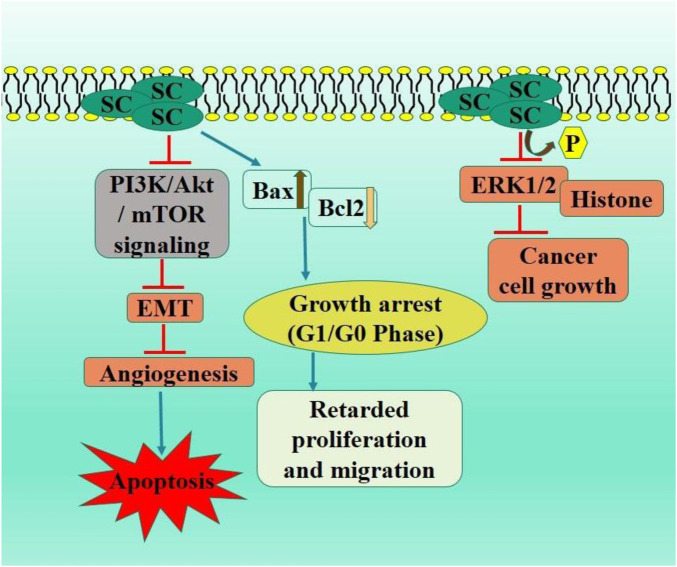
Diagrammatic illustration by which scutellarin induce apoptosis and growth arrest in non-small cell lung carcinoma.

He et al. further investigated the anticancer efficacy of iodine-125 (125I) and SC in A549 and H1975 cell lines. SC enhanced the apoptotic and antiproliferative effects generated by 125I via downregulated AKT/mTOR pathway. This study provides a robust basis for implementing this combined treatment in lung cancer care (He G. H. et al., 2021). Zhang et al. examined anticancer activity of SC and reported a substantial reduction in cell viability and apoptosis activation in SC treated A549 cells. SC also facilitated caspase activation, intracellular ROS generation, and the TGF-β1/smad2 signaling pathway activation (Figure 5). These investigations offer a robust basis for elucidating the mechanisms behind the anticancer potential of scutellarin in the therapy of NSCLC (Zhang et al., 2021).

### 3.5 Hepatocellular carcinoma

The predominant tumor associated with increased mortality rates is hepatocellular carcinoma, sometimes referred to as malignant hepatoma. The onset and advancement of HCC are precipitated by various variables, including hepatitis (B and C) infections, prolonged alcohol intake, fatty liver disease, age, metabolic disorders, and oxidative stress (122; Llovet et al., 2022). Sorafenib is the only medicine licensed by the US Food and medicine Administration for HCC treatment (Balogh et al., 2016). Numerous therapeutic modalities exist for HCC treatment, including adjuvant therapy, chemotherapy, and immunotherapy; nevertheless, these frequently result in various adverse effects (Kane et al., 2009; Kudo et al., 2017). However, current treatment options are insufficient due to the rising medication resistance and their toxicity. Numerous natural products assist in HCC prevention and treatment (Ikeda, 2019). Multiple signaling pathways are linked to the HCC prevention using plants and their active constituents (Rawat et al., 2018; Pandey et al., 2024). We have concentrated our review on describing the anticancer efficacy of SC in hepatocellular carcinoma. A study indicated substantial suppression of cell growth in HepG2 cells treated with SC. Furthermore, SC-treated cells displayed characteristic apoptotic morphology and reduced ROS production compared to untreated HepG2 cells. The STAT3 protein was reduced in SC treated HepG2 cells, resulting in downregulation of its transcriptional targets (Bcl-XL and Mcl-1). Consequently, SC suppressed proliferation and triggered apoptosis through the STAT3 signaling pathway, offering substantial evidence for using SC as an alternative therapy for liver cancer (Khan et al., 2024). In MHCC97-H and HepG2 and cells, SC presumably inhibited invasiveness by remodeling the cytoskeleton via EMT suppression process, likely due to downregulation of JAK2/STAT3 pathway. Altogether, these outcomes may offer novel clinical insights for the management of liver carcinoma (Xu and Zhang, 2013).

Isochlorate dehydrogenase 1 (IDH1) is a crucial metabolic enzyme involved in the synthesis of α-ketoglutarate (α-KG), which possesses anticancer properties and is regarded as having potential antitumor effects. In an *in vivo* study, IDH1 activation by SC significantly activated the tumor immune microenvironment, increased α-KG level in tumor tissue, and downregulated HIF1α signaling pathway. This study illustrated the suppressive impact of IDH1-α-KG-HIF1α on HCC cell proliferation. It assessed the inhibitory effect of SC, first IDH1 small molecule agonist, offering a reference for cancer immunotherapy targeting activated IDH1 (Liu et al., 2019).

**FIGURE 6:**
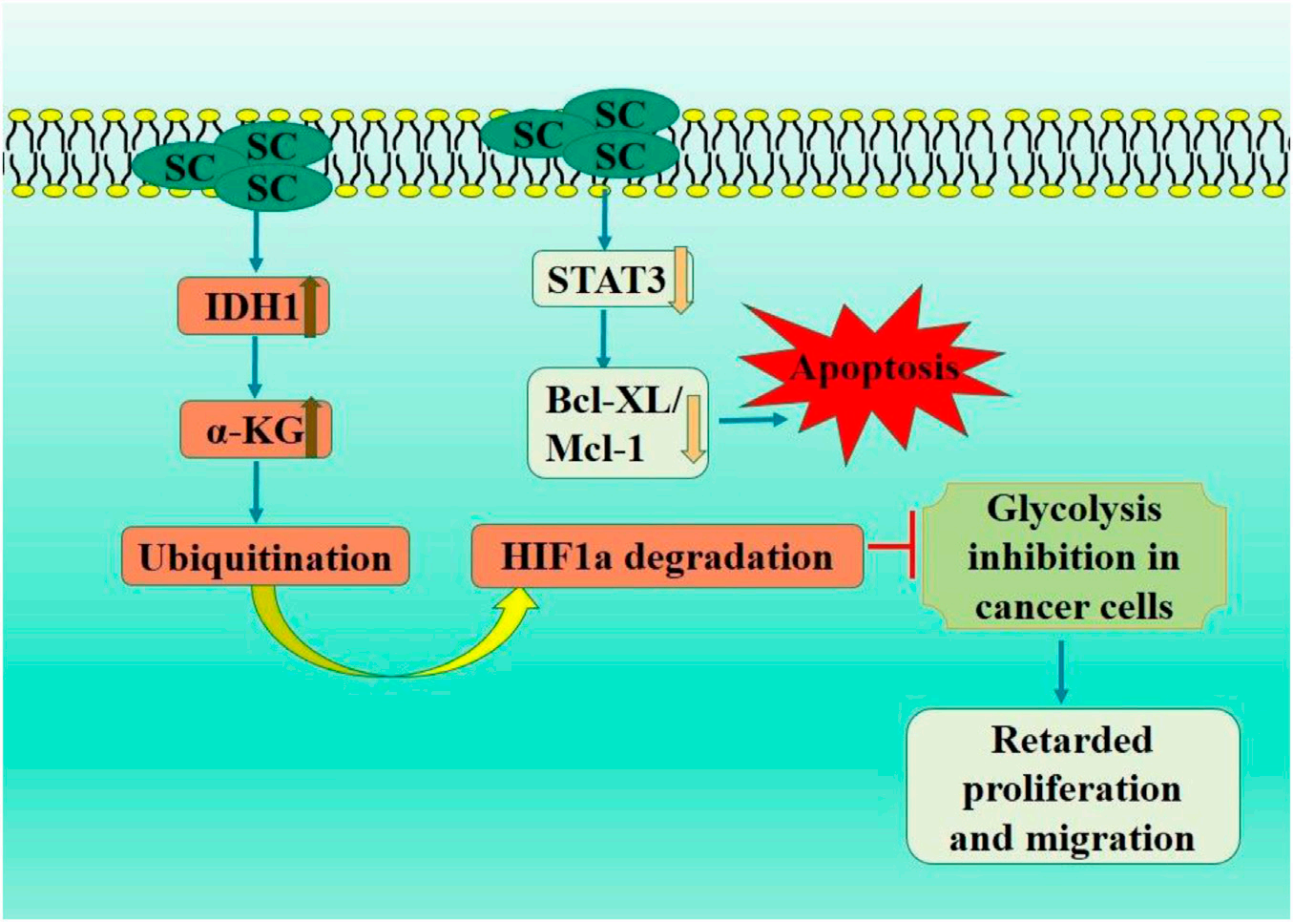
Diagrammatic illustration by which scutellarin induce apoptosis and growth arrest in colorectal carcinoma.

Ke et al. showed a substantial decrease in cell growth, migration, and invasion in SC treated HepG2 and HCC cells (Cui et al., 2024). Scutellarin therapy markedly reduced STAT3 and girdin expression levels and STAT3 and Akt phosphorylation in HCC cells. Moreover, overexpressed girdin induction entirely negated the inhibitory effects of SC on Akt phosphorylation and HCC cells invasion. SC impeded HCC cell metastasis and invasion via downregulated STAT3/Girdin/Akt signaling pathway (Ke et al., 2017) (Figure 6).

There are several other studies that reported the anticancerous effects of scutellarin in other carcinomas. Table 1 briefly describes their origin and mechanism of action in several human carcinomas.

**TABLE 1:** Anticancer potential of scutellarin and mechanism of action in various carcinomas.

Cancer type	Cells	Mode of action of scutellarin	References
Osteosarcoma	143B and U2OS cells	Reduced osteosarcoma cell growth Increased cell apoptosis and EGR1 expression Suppressed expression of LINC00857	Han et al. (2021)
Multiple Myeloma (MM)	Xenograft mouse model of MM	Reduced MM progression Combined treatment with bortezomib effectively enhanced the MM tumor progression Reversed chemo resistance and induced apoptosis in MM cells	Li et al. (2020)
Bladder Cancer	BC Cells	Inhibited hypoxia-induced BC cell migration and invasion (*in vitro* and *in vivo*)	Lv et al. (2019)
Skin Cancer	A375 cells	Inhibited viability and effectively suppressed tumor cell migration and adhesion via EMT suppression, angiogenesis Inhibited PI3K/Akt/mTOR signaling pathway	Li et al. (2019)
	K562 cells	Upregulated Bax and downregulated Bcl-2 Suppressed cell migration and invasion Induced G0/G1 cell cycle arrest Reduced expression of p-MEK1/2, p-ERK1/2, and p-Raf	Bao et al. (2020)
	T-LAK cell-Originated Protein Kinase (TOPK) (RPMI7951 cells)	Inhibited the proliferation and colony formation Inhibited phosphorylation levels of ERK1/2 and H3	Mu et al. (2021)
Cervical cancer	HeLa cells	Inhibited cellular growth and glycolysis via inhibiting pyruvate kinase M2 activity and directly targeting pyruvate kinase M2 (PKM2) Inhibits cytosolic activity to reduce glycolytic metabolism	You et al. (2017)

## 4 Scutellarin as a novel agent for cancer chemotherapy to overcome chemotherapeutic drugs induced resistance

Numerous platinum compounds including carboplatin and cisplatin (CP) have been extensively employed in the treatment of several solid malignancies (Giaccone, 2000). Cisplatin (Ist line anti-cancer agent) has been employed for the treatment of numerous malignancies (Ghosh, 2019; Romani, 2022). CP treatment leads to chemoresistance, which results in therapeutic failure (even with high initial response rate). Resistance to CP is ascribed to three molecular mechanisms including augmented drug inactivation, enhanced DNA repair, and modified cellular accumulation (Yue et al., 2023; Florea and &Büsselberg, 2011). Using natural products to combat chemotherapeutic drug resistance in cancer treatment has gained significant attention by exhibiting their capacity to reverse chemoresistance (Talib et al., 2021; Guo et al., 2017). In A549/DDP cells, scutellarin has demonstrated the ability to sensitize these cells to CP via augmenting autophagy and apoptosis. Experimental investigations revealed enhanced cisplatin-induced caspase-3-dependent apoptosis via activating extracellular signal-regulated kinases (ERK)-mediated p53 pathway (Sun C. et al., 2018). Moreover, scutellarin downregulated the expression of c-met and p-AKT while enhanced CP induced cytotoxic autophagy. c-Met deficiency lead to decreased p-AKT levels while suppressed autophagy (p-AKT or c-Met-enhanced) in A549/DDP cancer cells. Notably, the impairment of autophagy diminished the synergistic effect of this combination. Coadministration of CP and SC significantly decreased tumor size relative to CP monotherapy. SC markedly diminished CP induced toxicity in tumor-bearing mice. This study elucidated the distinct function of SC in counteracting CP resistance via autophagy and apoptosis, proposing that the combination of CP and SC could represent an innovative treatment approach for NSCLC patients (Sun C. et al., 2018).

**FIGURE 7:**
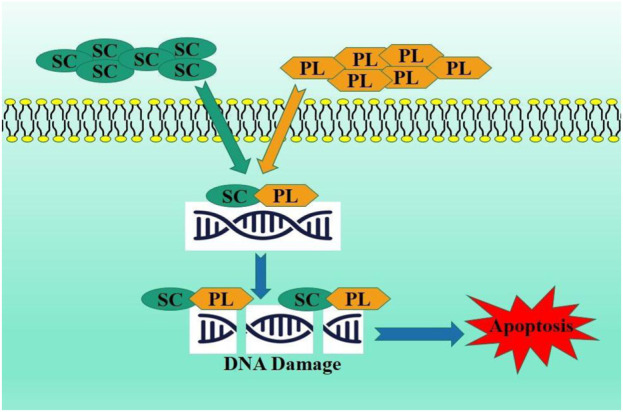
Combination of scutellarin and platinum compounds as potent therapeutic strategy.

Platinum resistance has become significant obstacle in the treatment of ovarian cancer (Yang et al., 2022). Scutellarin enhanced the antitumor efficacy of CP against ovarian carcinoma. The combined administration of SC and cisplatin (CP) increased apoptosis in ovarian cancer cells by elevating formation of platinum-DNA adducts and the Bax/Bcl-2 ratio. Scutellarin formed a compound with cisplatin, inhibiting BamH1 digestion of pBR322 plasmid DNA more effectively, indicating that their association may generate a more significant conformational change in the DNA, ultimately leading to DNA strand breaks and improved apoptotic signaling cascade activation. This study robustly demonstrated that SC functions as a possible sensitizer to CP treatment, suggesting that the combination of SC and CP may embody a unique therapeutic approach to address platinum resistance in ovarian cancer (Xie et al., 2019) (Figure 7).

Oxaliplatin-resistant colorectal cancer cells (OR-HT29 and OR-SW480) were obtained via prolonged exposure to oxaliplatin, demonstrating significantly reduced sensitivity and an elevated glucose metabolism rate compared to their parental cells (HT29 and SW480) (Sun et al., 2021). Nonetheless, the combination with scutellarin ostensibly resensitized oxaliplatin-resistant colorectal cancer cells to cytotoxicity, inhibited PKM2 activity, and consequently diminished ATP production, thereby sensitizing oxaliplatin-induced mitochondrial apoptotic pathway in both of the cancerous cells (OR-HT29 and OR-SW480 cells) (Sun et al., 2021).

In addition to its anti-tumor properties, SC has demonstrated that combinatorial therapeutics with it can mitigate the adverse efficacies of the primary medication. SC pretreatment mitigated CP induced histological damage in renal tissue and reduced expression levels of BUN and creatinine. Scutellarin reduces kidney inflammation via reducing expression levels of pro-inflammatory cytokines (IL-6 and TNF-α). Likewise, the administration of SC inhibited CP induced apoptosis via reducingp53 expression levels, Bax/Bcl-2 ratio, cleaved caspase-3, and PARP. Animals treated with scutellarin exhibited potent reduction in the activation of p38, STAT3, JNK, and ERK and induced by CP in kidneys. The findings prove that SC is a novel renoprotective agent against CP-induced renal injury (Sun et al., 2021). Diosbulbin B (diterpene lactone) is a conventional Chinese remedy for thyroid disorders and has demonstrated a significant anti-cancer activity *in vivo*. Diosbulbin B exhibits significant anti-cancer efficacy in clinical applications; nevertheless (with substantial hepatotoxicity) (Guan et al., 2017). In a combinatorial therapy involving SC and Diosbulbin B, SC has been demonstrated to mitigate Diosbulbin B-induced hepatic injury via diminishing NF-κB-mediated hepatic inflammation and alleviating oxidative stress in the liver, resulting in significantly reduced serum levels of ALP, AST, and ALT. Moreover, SC enhanced the antitumor efficacy of Diosbulbin B in S180 tumor-bearing mice (Niu et al., 2015). Thus, the combination therapy of platinum compounds and SC has demonstrated efficacy in mitigating resistance to platinum compound treatment, highlighting the advancement and utilization of natural product-based formulations of platinum compounds as an innovative therapeutic approach to combat human cancers.

Tumor tissues have higher blood pressure and viscosities with disorganized tumor vasculature, which makes drug administration difficult (Martin et al., 2019). Through non-cavitating mechanical efficacies and cavitation-induced jet stream, sonication enhances membrane permeability which ultimately raises intracellular uptake and concentration of desired drug at specific tumor sites (Lyons et al., 2023). Sonication may enhance chemotherapy’s efficacy by augmenting medication distribution to tumor sites and enhancing the permeability of cancer cell membranes (Tsuru et al., 2012). *In vitro* and tumor-xenografted mice, a study employing SC and ultrasound treatment for human tongue carcinoma revealed that the combination therapy inhibited angiogenesis and metastasis and delayed tumor growth in comparison to SC alone and ultrasound-alone therapies (Li H. et al., 2013). SC combined with new anti-tumor drugs produced more effective therapeutic effects in addition to well-known ones. The mechanism underlying these outcomes might be connected to inhibit PSEN1/PI3K-AKT signaling axis. For instance, SC and its combination with C_18_H_17_NO_6_ (caffeoyl tyrosine) decreased the proliferation and migration of glioma cells, thereby leading to cell death (Wang et al., 2023; Tang et al., 2019; He et al., 2019). These findings demonstrate that scutellarin can provide potent anti-tumor responses when combined with conventional and new chemotherapeutic drugs. Additionally, a combined treatment scutellarin with baicalin acted synergistically to increase glucose uptake in adipocytes via differential regulation on AMPK and Akt activity. These findings provided insight that multicomponent herbal medicines may act synergistically on multiple targets (Yang L. L. et al., 2017).

## 5 Conclusion

Scutellarin (flavonoid) displayed numerous pharmacological and biological potential, including anti cancerous, anti-coagulation, anti-microbial, anti-oxidant, and anti-rheumatoid. Scutellarin (SC) modulates in numerous cell signaling molecules (mTOR, STAT3, NF-κB, and AKT) and suppresses their associated signaling pathways (HIPPO-YAP, PI3K/Akt/mTOR, Wnt/β-catenin, and Raf/MEK/ERK) in cancer cells. These modulations in cell signaling pathways then initiate different cellular processes by which SC induces and suppresses genes associated with mediating tumor cell angiogenesis, proliferation, metabolism, and metastasis. Additionally, scutellarin influences the tumor microenvironment, suppressing excessive inflammation that allows tumor cells to proliferate and boosting immune cell responses. Most research on anti-tumor action of SC has been done *in vitro*, while some studies have also used animal models. Consequently, its anti-tumor properties *in vivo*, especially in humans, are unknown. In order to ascertain whether scutellarin can be incorporated into treatment plans, it is recommended that it be evaluated in clinical trials. No study has been reported to display the synergistic efficacy of scutellarin with other natural compounds for cancer management. Thus there is a strong need to investigate its anti cancer potential with other natural compound. These findings may facilitate the development of effective and promising cancer therapeutic regimens if implemented in clinical practice.

## Abbreviations

SC, Scutellarin; MRP3, Multidrug Resistance-Associated Protein 3; BCRP, breast cancer resistance protein; PLGA, Poly(lactic-co-glycolic acid; FA, Folic acid; NO, Nitric oxide; PCD, polymer cyclodextrin; NCS, nanocarrier system; CDKs, cyclin-dependent kinases; CDKIs, cyclin-dependent kinases inhibitors; PTEN, Phosphatase and tensin protein homolog; CAC, colitis-associated colorectal cancer; Tregs, regulatory T lymphocytes; TNFR2, Tumor necrosis factor receptor 2; BCSCs, breast cancer stem cells; (IDH1), Isochlorate dehydrogenase 1; (α-KG), α-ketoglutarate; PKM2, Pyruvate Kinase M2; BUN, Blood Urea Nitrogen; PARP, poly (ADP-ribose) polymerase; STAT3, signal transducer and activator of transcription 3; JNK, c-Jun N-terminal kinase; ERK, extracellular signal-regulated kinase; ALP, alkaline phosphatase; AST, aspartate aminotransferase; ALT, alanine aminotransferase; HIPPO-YAP, Hippo-Yes-associated protein signaling pathway.
